# In vitro comparison of performance including imposed work of breathing of CPAP systems used in low-resource settings

**DOI:** 10.1371/journal.pone.0242590

**Published:** 2020-12-03

**Authors:** Megan Heenan, Jose D. Rojas, Z. Maria Oden, Rebecca Richards-Kortum

**Affiliations:** 1 Rice 360°: Institute for Global Health, Rice University, Houston, Texas, United States of America; 2 Department of Respiratory Care, School of Health Professions, University of Texas Medical Branch, Galveston, Texas, United States of America; 3 Department of Bioengineering, Rice University, Houston, Texas, United States of America; University of New South Wales, AUSTRALIA

## Abstract

Respiratory distress due to preterm birth is a significant cause of death in low-resource settings. The introduction of continuous positive airway pressure (CPAP) systems to treat respiratory distress significantly reduced mortality in high-resource settings, but CPAP was only recently introduced in low-resource settings due to cost and infrastructure limitations. We evaluated pressure stability and imposed work of breathing (iWOB) of five CPAP systems used in low resource settings: the Fisher and Paykel bubble CPAP, the Diamedica baby CPAP, the Medijet nCPAP generator, and the first (2015) and second (2017) generation commercially available Pumani CPAPs. Pressure changes due to fresh gas flow were evaluated for each system by examining the relationship between flow and pressure at the patient interface for four pressures generated at the bottle (0, 3, 5, and 7 cm H_2_O); for the Medijet nCPAP generator, no bottle was used. The slope of the resulting relationship was used to calculate system resistance. Poiseuille’s law of resistance was used to investigate significant contributors to resistance. Resistance ranged from 0.05 to 1.40 $\frac{cm\;H_{2}O}{L/min}$; three CPAP devices had resistances < 0.4 $\frac{cm\;H_{2}O}{L/min}$: the Fisher and Paykel system, the Diamedica system, and the second generation Pumani bubble CPAP. The other two systems, the Medijet nCPAP generator and the first generation Pumani bCPAP, had resistances >1.0 $\frac{cm\;H_{2}O}{L/min}$. Imposed WOB was measured using an ASL5000 test lung to simulate the breath cycle for an infant (5.5 kg), a term neonate (4.0 kg), and a preterm neonate (2.5 kg). Imposed WOB ranged from 1.4 to 39.5 mJ/breath across all systems and simulated infant sizes. Changes in pressure generated by fresh gas flow, resistance, and iWOB differ between the five systems evaluated under ideal laboratory conditions. The available literature does not indicate that these differences affect clinical outcomes.

## Introduction

Nearly two million infants die each year in the first week of life; nearly half of these deaths arise from complications of preterm birth [1]. Respiratory distress syndrome (RDS) is one of the most significant short-term complications of preterm birth. Approximately 3–7% of all neonates suffer from respiratory distress, and it is 30 times more common in preterm than in term neonates. Additionally, rates of respiratory distress in low-resource neonatal units may be as high as 45% [2,3], and as many as 45% of deaths in neonatal units can be primarily attributed to RDS [3].

The World Health Organization and European Guidelines for Management of RDS recommend that respiratory distress in preterm newborns be treated with surfactants and continuous positive airway pressure (CPAP) [4,5]. In high-resource settings, survival rates for neonates with respiratory distress improved significantly after the introduction of CPAP [6,7]. Bubble CPAP (bCPAP), which provides pressure to a breathing circuit by immersing the distal end of the expiratory tubing in water, is a standard method of providing CPAP to neonates in respiratory distress. This is especially true in low-resource settings (LRS) where mechanical ventilation is not possible [8,9].

**Fig 1:**
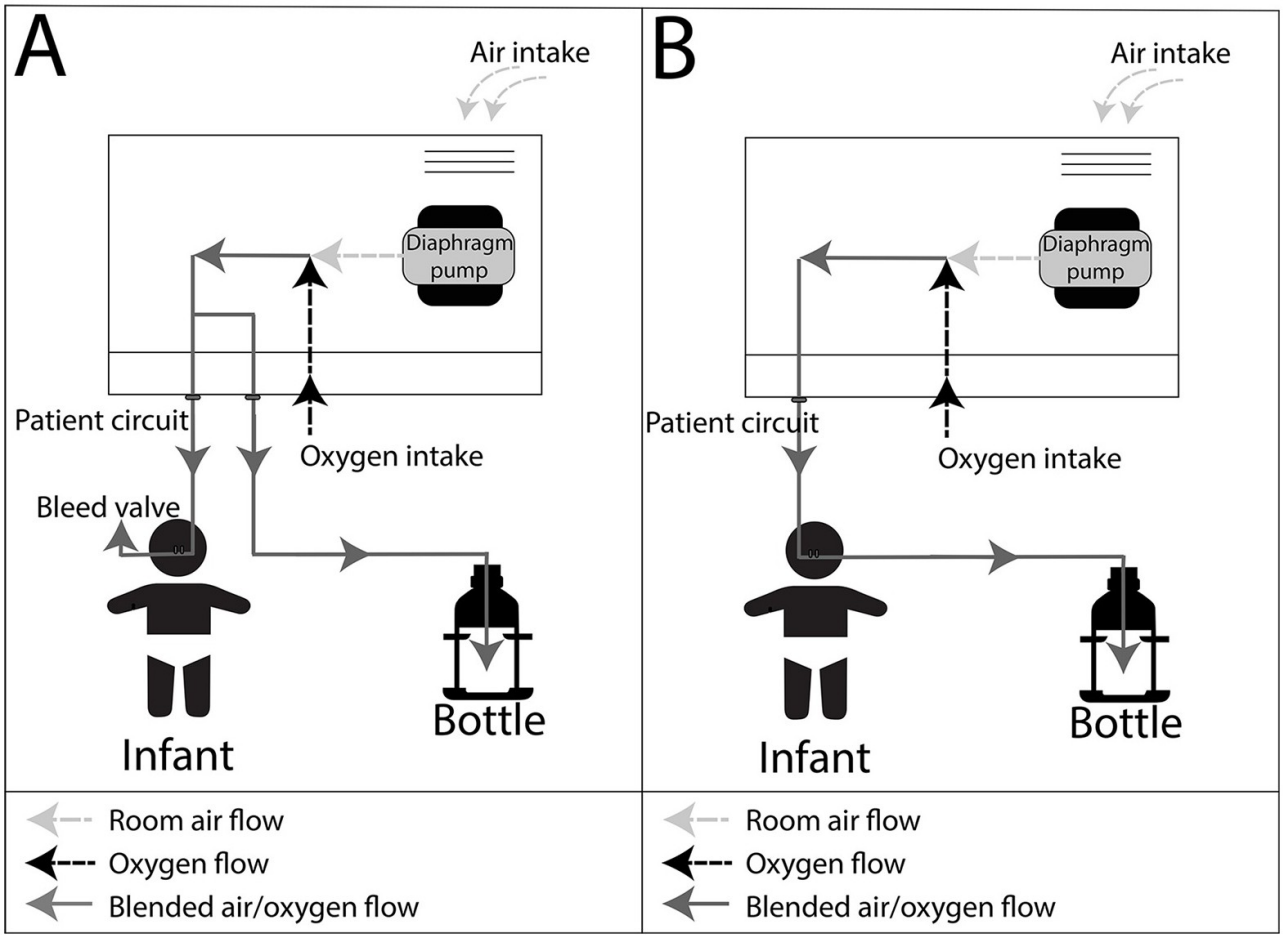
**(A)** First generation Pumani bCPAP device. In the first generation Pumani bCPAP device, air flows from the flow driver to the bottle and the patient interface in parallel. There is a bleed valve at the patient interface to prevent rebreathing of CO_2_. (**B)** Second generation Pumani bubble CPAP device. In the second generation Pumani bubble CPAP device, air flows from the flow driver to the patient interface to the bottle.

Low-resource settings present unique challenges for the design of medical devices, including lack of financial resources to purchase equipment, weak supply chains for consumables, inconsistent power, and harsh environmental conditions [10,11]. For this reason, a number of CPAP systems have been designed for use specifically in LRS, including the Diamedica baby CPAP and the Pumani CPAP [12–14]. The Diamedica baby CPAP includes a built-in oxygen concentrator for provision of oxygen to infants requiring CPAP in environments that do not have reliable oxygen supplies. The commercialized Pumani bCPAP device uses diaphragm pumps, rather than compressed air or medical gas supplies, to provide fresh gas flow. The first generation commercially available Pumani bCPAP (2015) included a bleed valve near the patient interface to prevent rebreathing (a modified Mapleson A circuit) [15]. The Pumani system was recently revised by the manufacturer; the second generation circuit of the Pumani bubble CPAP device (2017) delivers air first to the patient interface, then to the pressure-generating bottle (standard nCPAP circuit) (Fig 1) [16]. As part of the redesign, the diameter of the tubing connectors was also increased by 25%. The Medijet nCPAP generator uses a resistive system to eliminate the bubble pressure circuit; though not designed primarily for LRS, it has been used effectively in these settings [17]. The Fisher and Paykel bubble CPAP system, though intended for use in high-resource settings, has also been used in LRS [18]. These devices were selected for comparison as they have features that make them suitable for LRS and are currently used in these settings.

The purpose of this study was to compare pressures generated by fresh gas flow and imposed work of breathing (iWOB) of five CPAP devices used in low-resource settings.

## Materials and methods

We measured pressure generated by fresh gas flow and imposed work of breathing in a laboratory setting for five CPAP systems: Fisher and Paykel bubble CPAP (Fisher and Paykel, USA), Diamedica baby CPAP (Diamedica, UK), Medijet disposable nCPAP generator (medin Medical Innovations, DE), first generation commercially available Pumani bCPAP (Hadleigh Health Technologies, USA), and second generation commercially available Pumani bubble CPAP (Hadleigh Health Technologies, USA).

**Fig 2:**
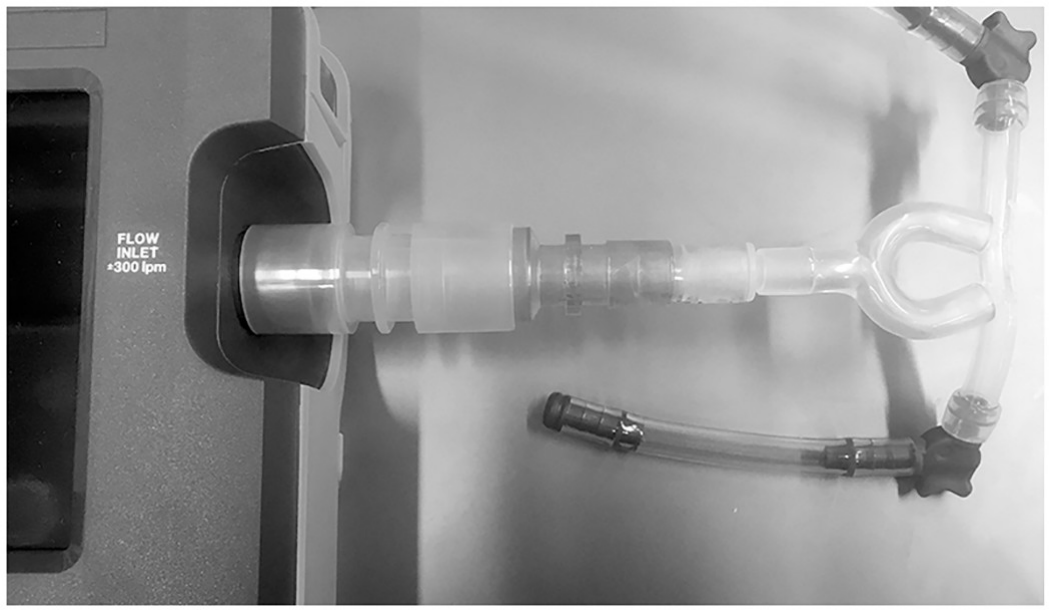
Prongs connected to the airway inlet of the VT650 gas flow analyzer. Prongs were connected to the device using a silicone Y-connector.

Flow rate was fixed using a flow meter for fresh gas. Pressure measurements were recorded using a Fluke VT650 Gas Flow Analyzer (Fluke Biomedical, USA) which was factory calibrated to meet manufacturer specifications (±0.5%) prior to data collection. Work of breathing was measured using an ASL 5000 Breathing Simulator with 3L cylinder (IngMar Medical, USA). The ASL system is calibrated annually to ensure pressure and volume accuracy. Pumani prongs (size 4) were used for all CPAP systems except the Medijet; for the Medijet, the interface was connected directly to both measurement devices. Prongs and the Medijet device were connected to the airway inlet (capable of measuring flow, pressure, volume and O_2_ concentration) of the Fluke VT650 using a silicone Y-connector (Fig 2).

### Pressure changes in response to fresh gas flow and system resistance

System resistance was evaluated by systematically increasing the fresh gas flow from 5–10 L/min and measuring the pressure at the patient interface. For all but the Medijet nCPAP system, the expiratory limb was submerged to 0, 3, 5, and 7 cm H_2_O as described in the user manuals of each device, while flow rates were increased from 5–10 liters per minute (L/min) at intervals of 1 L/min. For the Medijet nCPAP generator, pressure is provided solely through resistance, and therefore, there is no submersion of the expiratory limb. End expiratory pressures were recorded for each pressure/flow combination for one minute each. Pressures were recorded at a sampling rate of 1 Hz.

The first 10 and last 10 seconds of each recording were discarded and the mean and standard deviation of the remaining pressure measurements are reported. Resistance was calculated by determining the slope and 95% confidence interval of the best-fit line (least-squares method) of measured pressure vs. flow at 0 cm H_2_O of submersion depth. An F-test of significance was used to determine whether the slope of each line was significantly different than 0.

Resistances were compared against theoretical values calculated by Poiseulle’s law of resistance $R\;=\;\frac{8\eta l}{\pi r^{4}}$ where R = resistance; η = the dynamic viscosity of air; l = length of tubing; and r = radius of the tubing, connectors, and prongs. The dynamic viscosity of air at 22°C (η) was defined as 18.22 × 10^−6^ Pa-s [19]; length (l) and radius (r) were measured.

### Imposed work of breathing

A sinusoidal breath profile was used to simulate breathing of an infant (5.5 kg), a term neonate (4 kg), and a late preterm neonate (2.5 kg). Tidal volumes were estimated at 6 mL/kg body weight [20]. Infant breathing was simulated using a 32 mL tidal volume at 60 breaths per minute (bpm). Term neonate breathing was simulated using a 24 mL tidal volume at 67 bpm. Preterm neonate breathing was simulated using a 15 mL tidal volume at 67 bpm [20]. For all lung models and CPAP systems, the total resistance of the ASL/CPAP system is the sum of the resistances of both the ASL and CPAP individually. Resistance and compliance at the test lung were set to zero for the models used here. For all devices except the Medijet nCPAP generator, the expiratory limb was submerged to 6, 7, and 8 cm H_2_O, while flows were set at 6, 7, and 8 liters per minute (L/min). Each test lasted one minute. Breath-by-breath measurements (including imposed work of breathing) were calculated using the ASL 5000 software to determine the integral of the pressure-volume loop for each breath (1 Hz for infant model; 1.1 Hz for premature and neonatal infant models).

Imposed work of breathing measurements for the first ten and last ten breaths were discarded, resulting in 40 breaths remaining for the infant model and 47 breaths each for the term neonate and preterm neonate models. Mean and standard deviations of iWOB values were calculated. A paired t-test (α<0.05) was used to identify significant differences between systems. Statistical analysis was performed in MATLAB (Mathworks, Natick, MA) and SPSS (IBM, Armonk, NY).

## Results

### Pressure changes in response to fresh gas flow and system resistance

**Fig 3:**
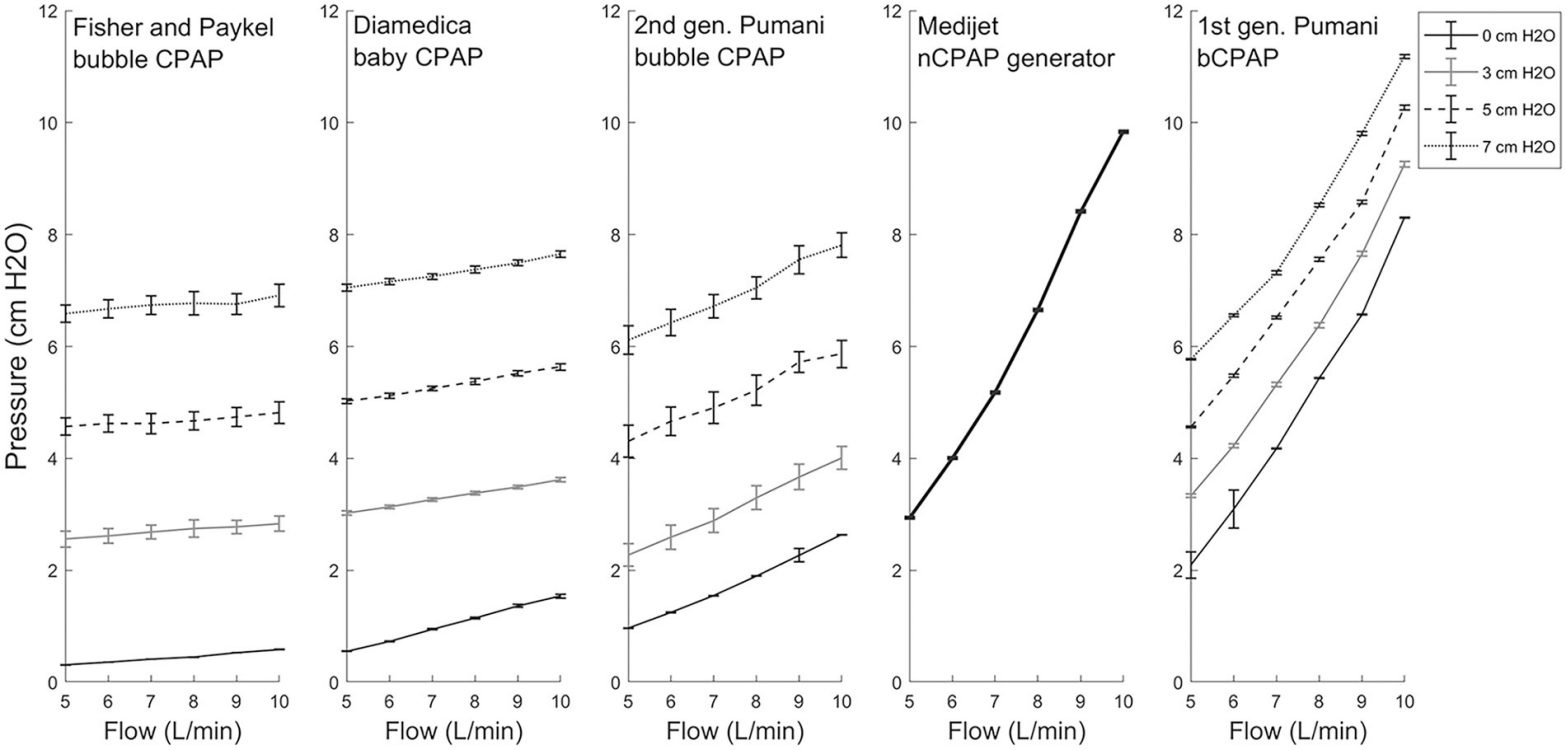
Pressure at patient interface vs. flow rate for (A) Fisher and Paykel bubble CPAP; (B) Diamedica baby CPAP; (C) second generation Pumani bubble CPAP; (D) Medijet nCPAP generator; (E) first generation Pumani bCPAP. Relationships are shown at 0 (solid black), 3 (solid gray), 5 (dashed black), and 7 (dotted black) cm of submersion in H_2_O. For the Medijet, only one value (solid black) is shown. Error bars represent one standard deviation. Data in S1 Table.

Pressure at the patient interface was plotted against applied flow for 0, 3, 5, and 7 cm of submersion for all but the Medijet nCPAP system (Fig 3). For the Medijet nCPAP generator, pressure is provided solely through pressure generation in the chamber, and therefore, there is no submersion of the expiratory limb.

Resistance of each system is calculated as the slope of each line from 5–10 L/min at 0 cm H_2_O and is shown in Table 1. For the Medijet system, resistance was calculated from the line indicating no externally applied pressure. All slopes were significantly different than zero (F-test, p < 0.001). Resistances range from 0.055 $\frac{cm\;H_{2}O}{L/min}$ (Fisher and Paykel bubble CPAP) to 1.40 $\frac{cm\;H_{2}O}{L/min}$ (Medijet nCPAP generator). The resistances of both the Diamedica baby CPAP and both Pumani systems fall in between these two values, at 0.20 $\frac{cm\;H_{2}O}{L/min}$ (Diamedica), 0.34 $\frac{cm\;H_{2}O}{L/min}$ (second generation Pumani) and 1.22 $\frac{cm\;H_{2}O}{L/min}$ (first generation Pumani).

**Table 1:** Resistance of CPAP systems at 0 cm H_2_O submersion depth.

CPAP system	Resistance ($\frac{cm\;H_{2}O}{L/min})$ at 0 cm H_2_O (95% ci)
Fisher and Paykel bubble CPAP	0.055 (0.054–0.056)
Diamedica Baby CPAP	0.20 (0.20–0.20)
2^nd^ generation Pumani bubble CPAP	0.34 (0.33–0.34)
Medijet nCPAP generator	1.40 (1.38–1.42)
1^st^ generation Pumani bCPAP	1.22 (1.20–1.24)

**Fig 4:**
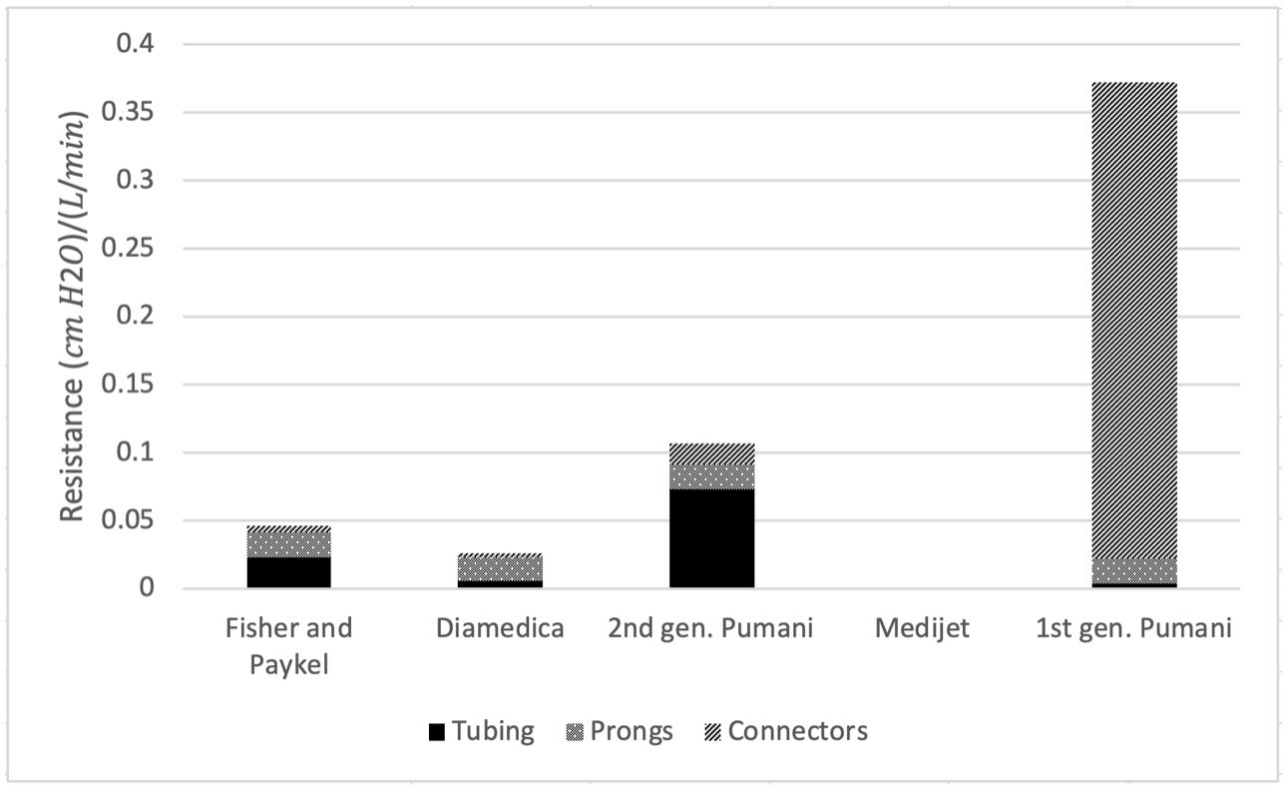
Theoretical contributions of tubing, prongs, and connectors to calculated resistance. Height of the bars indicates measured resistance of the expiratory limb for each CPAP system. Theoretical contribution of system tubing (solid), prongs (dotted), and connectors (diagonal lines) are shown. Data in S2 Table.

Contributions to resistance in the expiratory limb were examined using Poiseuille’s law of resistance. Diameters and lengths of tubing, prongs, and connectors for inspiratory and expiratory limbs of each system were measured. For the first generation Pumani bCPAP, where air flows in parallel to the bottle and patient, only the patient limb of the circuit was included here. Theoretical resistances are plotted in Fig 4.

### Imposed work of breathing

Imposed work of breathing (mJ/breath) for three representative flow/pressure combinations (6, 7, and 8 L/min; 8 cm H_2_O) is shown for the infant, term neonate, and preterm neonate for the Fisher and Paykel, Diamedica and first and second generation Pumani CPAP systems. iWOB for remaining pressure/flow combinations (6, 7, and 8 L/min; 6, 7 cm H_2_O) is shown in S1 Fig. For the Medijet nCPAP generator, pressure cannot be set independently; results are shown for the flow rates of 6, 7, and 8 L/min. A univariate ANOVA with Tukey post-hoc test was performed to identify whether differences in iWOB were significant across devices. All between-device differences in iWOB were statistically significant at the p<0.05 level (p<0.001) with the exception of Diamedica baby CPAP and Fisher and Paykel bubble CPAP with 6 L/min fresh gas flow for a preterm neonate (p = 0.078).

## Discussion

The CPAP systems evaluated here had resistances ranging from 0.05 to 1.40 $\frac{cm\;H_{2}O}{L/min}\;$ as determined by a linear fit of pressure vs. flow. Three CPAP devices had resistances < 0.4 $\frac{cm\;H_{2}O}{L/min}$: the Fisher and Paykel system, the Diamedica system, and the second generation Pumani bubble CPAP (2017). For these three devices, calculations of theoretical resistance showed that system resistance was generated primarily by the patient tubing, with contributions from connectors and prongs. The other two systems, the Medijet nCPAP generator and the first generation Pumani bCPAP (2015), had resistances >1.0 $\frac{cm\;H_{2}O}{L/min}$; calculations of theoretical resistance showed significant contributions to resistance from sources other than the patient circuit. One limitation of this analysis is that the linear relationship between pressure and flow holds for laminar flow but breaks down under conditions of turbulent flow, which are likely present here. Under these conditions, a second-degree polynomial may be a better fit, and thus this slope may capture additional features of the system (e.g. turbulence). The relationship between pressure and flow for the Medijet and first generation Pumani bCPAP shows some degree of non-linear resistance (Fig 3); however, here we used a linear fit for ease of comparison. Additionally, for the Medijet device in particular, resistance to patient breathing may differ from the resistance to fresh gas flow from the nCPAP generator inlet.

Poiseuille’s Law of Resistance was used to calculate theoretical resistance in each CPAP system. Although there are limitations to this calculation—In particular, that air flow through CPAP tubing may not be laminar—for one of the five systems evaluated here, theoretical resistance calculations account for > 90% of measured resistance. For the remaining three systems examined—Diamedica baby CPAP, second generation Pumani bubble CPAP and first generation Pumani bCPAP, the majority of resistance cannot be accounted for using Poiseuille’s Law; for these systems, it is likely that turbulence accounts for the remainder of the contribution. For the first generation Pumani bCPAP, the majority of resistance is provided by the connectors—In particular, the bleed valve used to prevent buildup of CO_2_. Turbulence at the bleed valve likely accounts for the remaining resistance. For the Medijet system, this is because the system is designed as a resistance system; the resistance is generated in the form of the modified Benveniste valve. For the Diamedica system, the remaining resistance may be generated by turbulence in the CPAP straw; air escapes the straw though 16 small orifices.

The imposed work of breathing for the second generation Pumani bubble CPAP system was reduced by approximately half from that of the first generation Pumani bCPAP system. This reduction was significant (p < 0.001). The second generation Pumani bubble CPAP also reduces the iWOB below that of the Medijet nCPAP generator system [21,22]; it remains higher than that of the Diamedica and Fisher and Paykel bubble CPAP systems [22,23]. As iWOB is highly correlated with resistance, the significant reduction in resistance predicts this reduction in iWOB.

We compared our results for imposed WOB to those previously reported for the infant breath profile the first generation Pumani bCPAP, Diamedica, Neopuff, Fisher and Paykel and Medijet systems. Values match those reported in Falk et al. [21–23]; the iWOB reported by Falk for the Diamedica system is approximately 9 mJ/breath (compared to 9 mJ/breath reported here); the iWOB reported by Falk for the Medijet system is approximately 30 mJ/breath (compared to the 32 mJ/breath reported here); and the iWOB reported by Falk for the Pumani (2015) system is approximately 40 (compared to the 39 mJ/breath reported here). One outlying value is the iWOB of the Fisher and Paykel bubble CPAP system; our measured value was higher than that reported by Falk et al; the iWOB reported by Falk for the Fisher and Paykel system is approximately 7 mJ/breath (compared to 15 mJ/breath reported here). We note that our measurements for all three systems were performed using the same size nasal prongs (4.0 mm inner diameter). Falk et al. used 4.0 mm inner diameter prongs to measure the iWOB with the Fisher and Paykel system and 3.8 mm inner diameter prongs to measure the iWOB with the first generation Pumani bCPAP system. The iWOB measured for the Fisher and Paykel system is high even though the resistance of the system is low; the Diamedica baby CPAP device, which has similar resistance, has lower iWOB for the infant breath profile. The smaller neonatal and preterm neonate breath profiles are associated with comparable iWOB for the Fisher and Paykel and Diamedica devices. It is important to note that the outlying value for the Fisher and Paykel device was registered on the same experimental setup used consistently across all devices. Additionally, leakage at the prongs, while capable of reducing measured CPAP, would likely also reduce iWOB; this did not occur for these readings.

### Clinical significance

The clinical significance of differences in resistance and iWOB is uncertain [23]. In this study, resistance for all five systems was measured under ideal conditions with a perfect seal at the nasal prongs. In practice, small leaks at the nasal prongs are likely to reduce pressure stability of all systems [24]. Previous laboratory and clinical studies have shown that pressure delivered to the nasal prongs in bubble CPAP systems exceeds the immersion depth of the expiratory tubing, with the pressure overshoot increasing with the magnitude of flow [25]; this finding was replicated here (as shown in Fig 3). Pressures at the patient interface were highest at 10 L/min of flow for the Medijet (9.8±.20 cm H_2_O) and first generation Pumani bCPAP with a submersion depth of 7 cm (11.2±.13 cm H_2_O).

**Fig 5:**
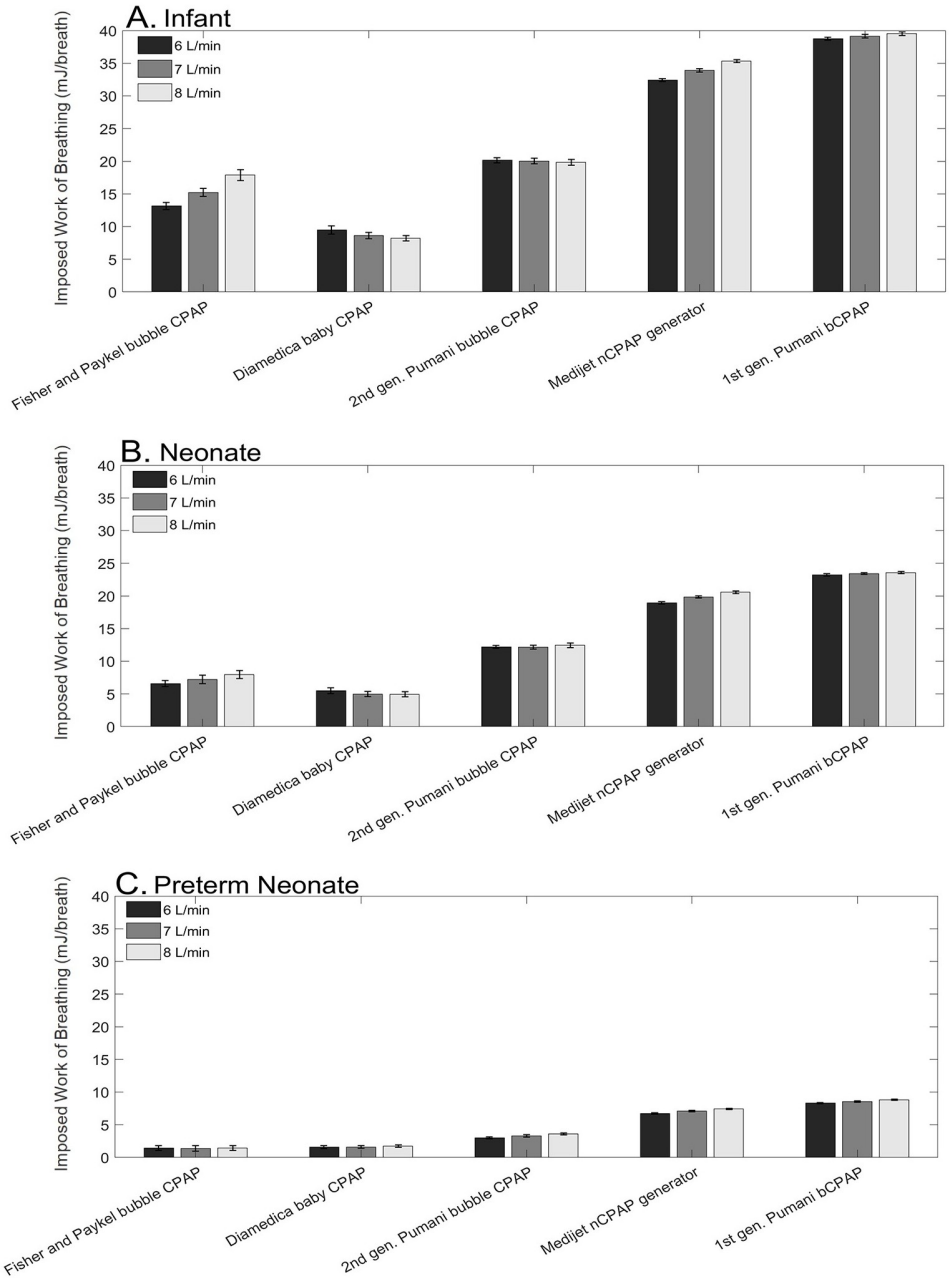
Imposed work of breathing for Fisher and Paykel bubble CPAP, Diamedica baby CPAP, 2^nd^ generation Pumani bubble CPAP, Medijet nCPAP generator, and 1^st^ generation Pumani bCPAP. Imposed work of breathing for the Fisher and Paykel, Diamedica and first and second generation Pumani CPAP systems at flow rates of 6, 7, and 8 L/min and 8 cm H_2_O submersion depth are plotted. For the Medijet nCPAP generator, pressure cannot be set independently; results for the flow rates of 6, 7, and 8 L/min are plotted. **(A)** Imposed work of breathing for breath profiles of an infant (5.5 kg) (32 mL lung volume, sinusoidal breath pattern, RR = 60); **(B)** Imposed work of breathing for a term neonate (4 kg) (24 mL lung volume, sinusoidal breath pattern, RR = 67); **(C)** Imposed work of breathing for a preterm neonate (2.5 kg) (15 mL lung volume, sinusoidal breath pattern, RR = 67). Error bars represent standard deviation. Data in S3 and S4 Tables.

There are no clinical guidelines for imposed work of breathing, and it varies significantly for commercially available systems, as shown in Fig 5. Due to the clinical efficacy of CPAP, randomized clinical trials—especially in low-resource settings—are rare and often small. Moreover, few studies have investigated the correlation between imposed work of breathing measured in laboratory-based studies and work of breathing observed during patient-device interaction [26]. However, a recent randomized clinical trial of 161 preterm neonates compared the duration of CPAP need and possible complications of two CPAP systems: the Medijet and Fisher and Paykel bubble CPAP systems [17]. There were no statistically significant differences in any of the study outcomes (p > 0.05) for any metric measured, despite the fact that laboratory measurement of the iWOB for the Medijet system is approximately 3-fold higher than that of the Fisher and Paykel bubble CPAP system [17]. Another study of 32 patients found that continuous-flow nCPAP imposes a greater work of breathing than variable-flow nCPAP. Despite the higher imposed WOB, patients receiving continuous-flow nCPAP did not exhibit changes in respiratory rate, thoraco-abdominal asynchrony, or require additional oxygen [27]. When compared to other reports of mortality reduction associated with CPAP in low-resource settings, a pre-commercial prototype using the same circuit as the first generation commercially available Pumani bCPAP device developed in 2014 (prior to 2015 commercialization) performs similarly to other available bCPAPs, despite significantly higher iWOB as measured in the laboratory; a recent study in Tanzania of the first generation Pumani bCPAP device also showed similar improvements in survival [6,28].

Although there is scant evidence to demonstrate that system resistance and imposed WOB are clinically significant, laboratory-based performance metrics such as these may help providers select devices appropriate for their clinical setting. This information may also help manufacturers improve device performance by identifying significant contributors to system resistance: for example, tubing diameter is a major contributor to resistance, and also predicts connector size. As resistance varies with the fourth power of diameter, small changes in diameter can result in significant improvements in resistance and iWOB. While tubing length is also an important contributor to overall resistance, small changes in tubing length have only a proportional impact on resistance. In addition, differences in laboratory performance may indicate areas where larger clinical trials are needed.

Another area of interest is CPAP provided during or immediately after resuscitation. For term infants in high resource settings, there is no current recommendation for CPAP at birth [29,30] and available evidence suggests that there is increased risk of air leak and pneumothorax [31,32]. Additional clinical trials may be needed to determine if additional device performance criteria are necessary for this use case.

## Conclusions

Laboratory testing under ideal conditions when no leaks are present at the patient interface shows that commercially available CPAP systems differ in resistance and iWOB. There are no available data to suggest that these differences affect clinical outcomes.

## Supporting information

S1 Fig Imposed work of breathing at flow rates of 6, 7, and 8 L/min and 6, 7, and 8 cm H_2_O submersion depth. Left: 8 cm H_2_O (shown in results); middle: 6 cm H_2_O; right: 7 cm H_2_O.
(TIF)

S1 Table Pressure at patient interface at fixed flow rates. (XLSX)

S2 Table Measurements of tubing length and diameter used to calculate theoretical resistance. (XLSX)

S3 Table Work of breathing at flow rates of 6, 7, and 8 L/min and 8 cm H_2_O submersion depth. For the Medijet nCPAP generator, pressure cannot be set independently; results for the flow rates of 6, 7, and 8 L/min were measured using ASL 5000 Breathing Simulator.
(XLSX)

S4 Table Tidal volumes and mean, minimum, and maximum airway pressures at 6, 7, and 8 L/min fresh gas flow and 8 cm H_2_O submersion depth measured using ASL 5000 Breathing Simulator. (XLSX)
