# Supplementary figures and images for: In vitro comparison of performance including imposed work of breathing of CPAP systems used in low-resource settings
Source: PLoS One. 2020 Dec 3;15(12):e0242590. doi: 10.1371/journal.pone.0242590 (PMC7714113; doi:10.1371/journal.pone.0242590)

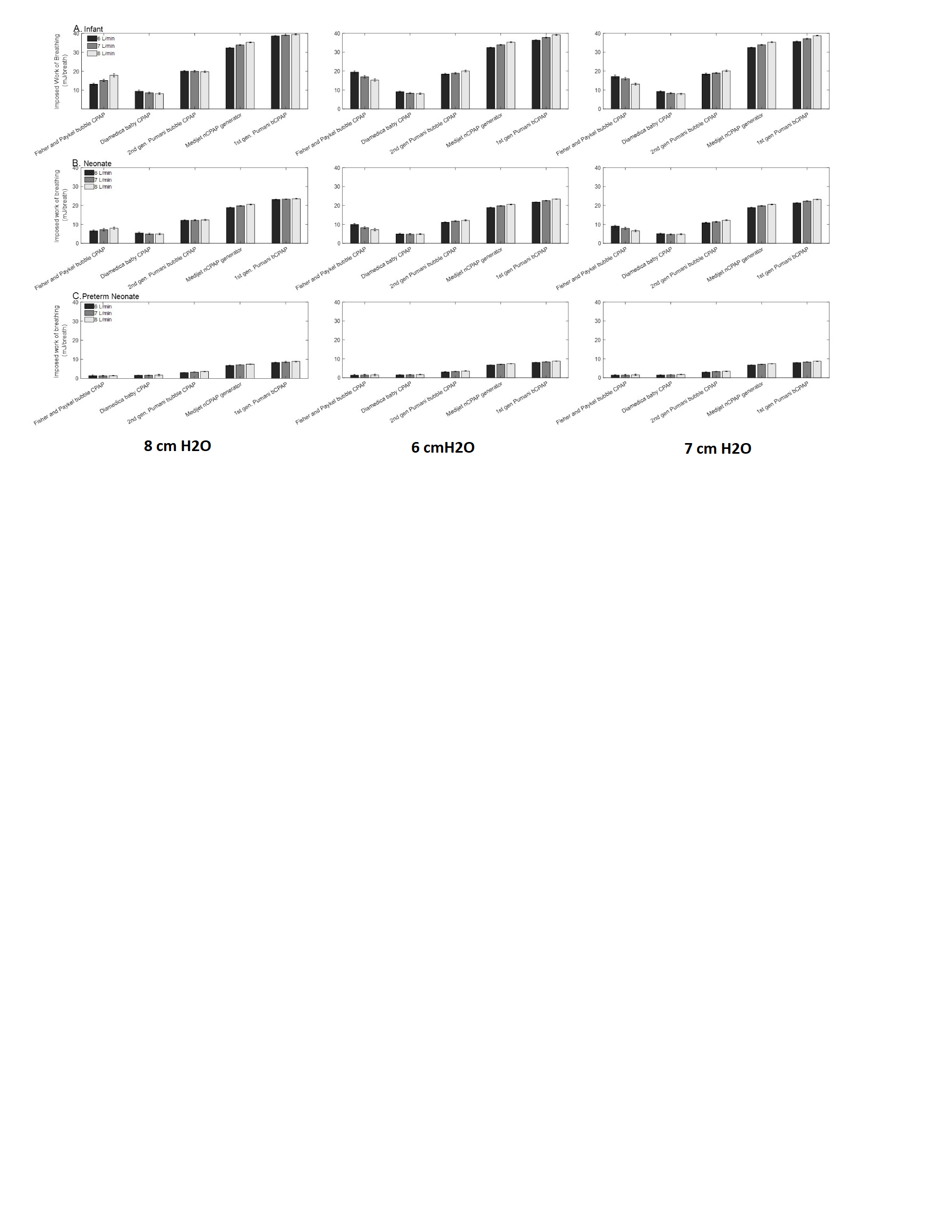

Supplement: S1 Fig — Left: 8 cm H2O (shown in results); middle: 6 cm H2O; right: 7 cm H2O. (TIF) [file pone.0242590.s001.tif]
